# Clinical Efficacy of Cemented Versus Screw‐Retained Zirconia Single Crowns: A Systematic Review and Meta‐Analysis

**DOI:** 10.1155/ijod/5497447

**Published:** 2026-06-12

**Authors:** Frank Mayta-Tovalino, Fran Espinoza-Carhuancho, Daniel Alvitez-Temoche, Ivan Calderon-Cortez, Miguel Cabanillas-Lazo, Joshuan J. Barboza, Adrian V. Hernandez

**Affiliations:** ^1^ Academic Department, Systematic Reviews and Meta-Analysis Unit (URSIGET), Vice-Rectorate for Research, Universidad San Ignacio de Loyola, Lima, Peru, usil.edu.pe; ^2^ Academic Department, Bibliometrics Evidence Evaluation and Systematic Reviews Group (BEERS) Human Medicine Career, Universidad Científica del Sur, Lima, Peru, cientifica.edu.pe; ^3^ Academic Department, Research, Innovation and Entrepreneurship Unit, Faculty of Dentistry, Universidad Nacional Federico Villarreal, Lima, Peru, unfv.edu.pe; ^4^ Academic Department, EVIDENTIA Research Group, Universidad Nacional Mayor de San Marcos, Lima, Peru, unmsm.edu.pe; ^5^ Academic Department, Neuroscience, Metabolism, Clinical and Health Effectiveness Research Group (NEMECS), Universidad Científica del Sur, Lima, Peru, cientifica.edu.pe; ^6^ Medicine Program, School of Health Sciences, Universidad Peruana de Ciencias Aplicadas, Lima, Peru, upc.edu.pe; ^7^ Health Outcomes, Policy and Evidence Synthesis (HOPES) Group, University of Connecticut School of Pharmacy, Storrs, Connecticut, USA, uconn.edu

## Abstract

**Background:**

The choice between cemented and screw‐retained zirconia single crowns remains a matter of debate, especially concerning long‐term biological and prosthetic outcomes.

**Objective:**

This systematic review and meta‐analysis aimed to compare the clinical and mechanical performance of cemented versus screw‐retained zirconia single crowns.

**Methods:**

An extensive search of PubMed, Embase, Web of Science, and Scopus was performed until July 2025. Only randomized controlled trials (RCTs) that compared cemented to screw‐retained zirconia single crowns were eligible for inclusion. Primary outcomes were marginal bone level (MBL) changes, bleeding on probing (BoP), and plaque index (PI). Secondary outcomes included probing depth (PD), modified PI (MPLI), width of keratinized (KT) mucosa, and mechanical complications (i.e., fracture and loosening). Risk of bias was evaluated using the Cochrane RoB 2.0 tool and certainty of evidence (CoE) was assessed with GRADE methods.

**Results:**

Nine RCTs involving 298 patients were included. Cemented zirconia single crowns probably result in a slight reduction in marginal bone loss compared with screw‐retained crowns (mean difference [MD] = –0.04 mm; 95% confidence interval [CI]: –0.08 to –0.00; *I* ^2^ = 0%; moderate CoE). Cemented crowns likely result in little to no difference in BoP, PI, PD, MPLI, and width of KT mucosa. Similarly, they likely result in little to no difference in mechanical complications such as fracture (risk ratio [RR] = 0.99; 95% CI: 0.82–1.19) and screw loosening (RR = 0.94; 95% CI: 0.50–1.77). Subgroup analyses confirmed these findings, except for MBL, where cemented crowns probably result in a slight reduction in longer follow‐ups and adult populations. Most included trials presented some concerns or high risk of bias, primarily related to outcome measurement and reporting.

**Conclusion:**

Cemented zirconia single crowns may lead to slightly less marginal bone loss than screw‐retained ones, but no meaningful differences were found in other biological or prosthetic complications. Current evidence remains limited, underscoring the need for well‐designed, long‐term RCTs to provide more definitive guidance.

## 1. Introduction

Dental implants have been defined as a highly effective therapeutic option for patients with partial tooth loss, especially in the case of single‐tooth gaps. Among the possibilities for prosthetic rehabilitation, implant‐supported crowns have established a new concept in modern dentistry, allowing for the long‐term rehabilitation of teeth in a functional and esthetic manner. Within this context, zirconia crowns have become increasingly popular due to their excellent biocompatibility, good durability, and esthetics like natural teeth [1, 2].

An interesting fact about zirconia is that it has a lower tendency to accumulate microbial plaques than other materials, which can promote the health of peri‐implant tissues. This can provide certain benefits in patients with a history of periodontitis or thin periodontal phenotype, where this type of inflammatory response could be more severe [3, 4].

In critical esthetic areas (e.g., the anterior region), several materials for implant‐supported crowns have appeared that improve the outcome for thin tissues. Single‐piece zirconia abutments would be particularly useful in situations with high esthetic demands, where they prevent the transparency effect that would be generated by a metal abutment under the gum, unlike metals. Some previous studies report similar success rates between zirconia and metal abutments (57.5%–93.3% and 57.1%–100%) for zirconia and titanium implants, respectively [5, 6].

The fracture resistance of these restorations depends on a multitude of factors: implant surface characteristics [7], surgical techniques, and prosthetic retention systems. Regarding the implant‐crown connection, there are two basic types of techniques: cementation and screw retention [8, 9]. The screw‐retained technique facilitates the removal of the prosthesis for maintenance, repairs, or adjustments, but the access hole left in a poorly aligned implant prosthesis can compromise esthetics and occlusion [10, 11].

The choice between the two methods for clinical practice, to the same level as that obtained with the traditional methods it challenges, is conditioned not only from an anatomical perspective but also from the professional’s learning, the accessibility of the implant, and even the future need for modifications. Therefore, solid scientific evidence is essential to establish standardized protocols; the goal will be to improve the final outcomes [12].

However, despite the progress made, there is still controversy over which technique provides the best long‐term results. Cemented crowns provide marginal sealing and simplicity in their manufacture, while screw‐retained crowns allow for maintenance and retrievability. In implants with marked inclinations, cementation on customized abutments, in addition to offering esthetic advantages, allows for the manufacture of crowns with greater precision and ease and better positioning in posterior areas [13, 14].

Despite this, this system is not without limitations, as the difficulty in removing excess cement is one of the drawbacks that can lead to peri‐implant complications if not done correctly. The accumulation of dental cement residue at the peri‐implant soft tissue interface is correlated with inflammation, bleeding on probing (BoP), or suppuration in the case of extraction with a brush [15, 16].

The purpose of this systematic review and meta‐analysis is to compare the clinical effectiveness of cemented versus screw‐retained zirconia single crowns, focusing on biological and prosthetic outcomes, in order to provide evidence‐based guidance for clinical decision‐making in implant‐supported restorations.

## 2. Methods

### 2.1. Study Design

This systematic review and meta‐analysis were conducted and reported following the Preferred Reporting Items for Systematic Reviews and Meta‐Analyses (PRISMA) statement [17]. The protocol was registered a priori in PROSPERO (Registration Number: CRD420261365207).

### 2.2. Search Strategy

A detailed search of the literature was conducted in the following electronic databases from inception to the search date: PubMed (Medline), Embase, Web of Science, and Scopus. The search included a combination of controlled vocabulary (e.g., MeSH and Emtree) and free‐text terms referring to zirconia single crowns, cemented, screw‐retained, and prosthetic outcomes (example search terms: “zirconia crown,” “single crown,” “cemented,” “screw‐retained,” “loosening,” “fracture,” “marginal bone loss,” “probing depth,” etc.). There were no language restrictions. The reference lists of studies that were included in this review and other relevant review articles were hand‐searched to identify additional records. The full search strategy for each database is provided in Supporting Information S1.

### 2.3. Eligibility Criteria

Eligible studies were randomized controlled trials (RCTs) that directly compared cemented versus screw‐retained zirconia single crowns in implant‐supported restorations. Participants included adult patients who received zirconia single crowns within these trials. Studies were required to report at least one of the prespecified clinical or mechanical outcomes, from which data could be extracted. Only RCTs with described methodologies and follow‐up periods were considered for inclusion.

Observational studies, case reports, case series, animal studies, in vitro studies, and review articles, along with any trials exploring restorative materials other than zirconia and restorative life, where single crowns were not the focus of the study, were also excluded. In some cases, studies that reported restorations of mixed or uncertain designs or studies that did not report sufficient data regarding the outcomes of interest or in the case of a specific outcome, were excluded.

### 2.4. Outcomes of Interest

The primary outcomes assessed were marginal bone level (MBL) changes, measured in millimeters as mean changes from baseline or between groups; BoP, reported as the percentage of sites or patients with bleeding; and plaque index (PI), evaluated with the scales applied in the original studies, generally as mean index scores. Secondary outcomes included probing depth (PD), measured in millimeters; modified PI (MPLI), recorded as mean index scores; and width of KT mucosa, also measured in millimeters. Mechanical complications were evaluated as dichotomous outcomes: fracture, reported as the number of fractured crowns relative to the total sample and expressed as risk ratios (RRs) or odds ratios; and loosening, defined as prosthesis or abutment screw loosening, reported as the number of events over the total.

### 2.5. Study Selection and Data Extraction

All records retrieved by the search were exported to reference management software and duplicates were removed. Two independent reviewers (Frank Mayta‐Tovalino and Daniel Alvitez‐Temoche) screened the titles and abstracts for eligibility (https://www.rayyan.ai/). Full texts of potentially eligible articles were obtained and assessed independently by the same two reviewers against the inclusion criteria. Disagreements at any stage were resolved by discussion and, when necessary, by consulting a third reviewer (Joshuan J. Barboza). Two reviewers independently extracted data using a piloted data‐extraction form. Extracted items included study identifiers (authors, year, country), study design, sample size, characteristics of participants (age, sex), type of restoration and implant system (if applicable), follow‐up duration, details of intervention and comparator (cement type, screw torque, occlusion, provisionalization), numerical results for all prespecified outcomes (means, standard deviations, change scores, number of events, and denominators), and information for risk of bias assessment. When necessary, the corresponding authors were contacted to request missing data or clarification. Any discrepancies in data extraction were reconciled by discussion or a third reviewer.

### 2.6. Risk of Bias Assessment

The risk of bias for RCTs was assessed independently by two reviewers using the Cochrane Risk of Bias 2.0 (RoB 2.0) tool. Domains evaluated included (1) bias arising from the randomization process; (2) bias due to deviations from intended interventions; (3) bias due to missing outcome data; (4) bias in measurement of the outcome; and (5) bias in selection of the reported result. Each domain was judged as “low risk,” “some concerns,” or “high risk,” and an overall risk of bias judgement was assigned to each study. Disagreements were resolved by consensus or by a third reviewer.

### 2.7. Data Synthesis and Statistical Analysis

Meta‐analyses were performed when reports with at least two comparable studies evaluated the same outcome. The continuous outcomes evaluated on the same scale—MBL, PD, and KT mucosa width—in millimeters were combined using mean difference (MD) with 95% confidence intervals (CIs). The outcomes that were reported on a different scale, plaque indices, were combined using standardized MD (SMD). Dichotomous outcomes, failure or loosening, were combined as the RR. A random‐effects model was applied with the inverse variance method with the Hartung–Knapp 95% CI adjustment when there were five trials or more per outcome. Heterogeneity was assessed using *χ* ^2^ and quantified using *I* ^2^ [18]. Publication bias was assessed with funnel plots and Egger’s test when the number of studies was ≥10. Analyses were performed with R version 4.5.1. Certainty of evidence (CoE) per outcome was rated using the GRADE methodology; CoE was rated as high, moderate, low, or very low and presented in a summary of findings (SoF) table [19].

### 2.8. Subgroup and Sensitivity Analyses

Subgroup and sensitivity analyses were conducted to explore potential sources of heterogeneity. Subgroup analyses were performed according to follow‐up duration (<5 years vs. ≥5 years) and by population type (adult, mixed/“both,” or older patients). Sensitivity analyses involved excluding studies judged at high risk of bias and reanalyzing the data with fixed‐effect models to assess the robustness of the findings. Additionally, leave‐one‐out analyses were performed to evaluate the influence of individual studies on the overall effect estimates. A *p*‐value for interaction <0.1 was considered statistically significant for subgroup comparisons (Supporting Information S2).

## 3. Results

### 3.1. Selection of Studies

**Figure 1:**
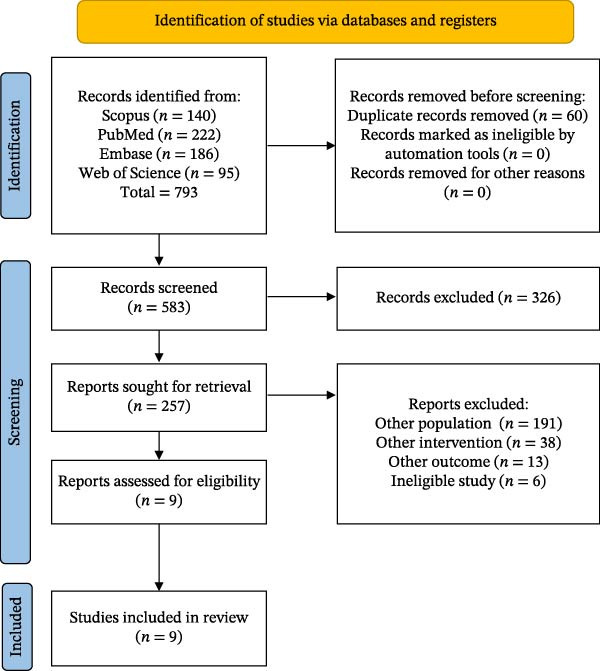
PRISMA flow chart of the study selection process.

After reviewing the full texts, 257 articles were excluded for various reasons. Most of them involved populations that did not match our criteria (*n* = 190), while others assessed different interventions (*n* = 48). A smaller number of studies were excluded because they reported outcomes outside the scope of our research question (*n* = 7), used study designs that did not meet eligibility requirements (*n* = 6), or provided insufficient data for analysis (*n* = 6). In the end, only nine RCTs [20–28] met all the inclusion criteria and were included in the meta‐analysis. The complete study selection process is summarized in Figure 1.

### 3.2. Characteristics of Included Studies

Table 1 described characteristics of included RCTs. Studies were from a sufficiently large range of countries, thus presenting a global interest to compare cemented and screw‐retained zirconia single crowns. Long‐term evidence was represented by Italy, as the authors conducted a 10‐year prospective randomized trial with strict inclusion and exclusion criteria [20]. Switzerland had the greatest number of trials, as noted by Heierle et al. [21], Kraus et al. [22], Lamperti et al. [24], and Thoma et al. [26], with follow‐up durations ranging between 6 months and 5 years. With these studies [20–22, 24, 26], the focus remained on the single tooth in the esthetic zone. Furthermore, Germany and Estonia provided study data and one split‐mouth clinical trial from Weigl as an additional resource for anterior and posterior single crowns. Only one study from China was noted for angulated screw‐retained zirconia crowns versus cemented zirconia crowns over a 1‐year randomized study [25]. Patient sample sizes ranged from 22 to 60 patients, with most patients between the ages of 30 and 50 years old and a generally balanced gender representation reported when indicated. Across all countries, studies consistently exclude patients with systemic diseases, poor oral hygiene, or heavy smoking habits, ensuring clinically comparable cohorts. Implant systems varied, with Straumann and Dentsply Sirona being the most common in Swiss and Italian trials, Nobel Biocare in China, and Ankylos in the German‐Estonian study. Retention methods were balanced, with each trial including both cemented and screw‐retained crowns, typically fabricated on customized CAD/CAM zirconia abutments. Prosthetic complications such as minor chipping, screw loosening, and occasional abutment fractures were observed across countries, though their frequency remained relatively low. Biological complications, including peri‐implant mucositis and isolated cases of peri‐implantitis, were reported mainly in Swiss trials, while Chinese and German/Estonian cohorts documented stable peri‐implant health. Soft tissue outcomes, measured through indices such as BOP, PD, and PES/WES, remained comparable between groups across all geographic contexts, supporting the overall clinical consistency of the findings.

**Table 1:** Characteristics of included randomized controlled trials.

Study, year [reference]	Country	Study design	Follow‐up	Sample size (patients)	Age	Gender distribution	Inclusion criteria	Exclusion criteria	Implant system used	Abutment material	Retention type	Number of cemented crowns	Number of screw‐retained crowns	Prosthetic complications	Biological complications	Soft tissue outcomes
Amorfini et al. 2018 [20]	Italy	10‐year randomized prospective study	10 years	32	ZrC: 48.3 (25–76), FCA: 47.6 (24–73)	ZrC: 7M/9F, FCA: 6M/10F	>21 years, no relevant medical disease, no periodontal disease, 1 failed tooth anterior maxilla, 2 intact adjacent teeth, contralateral tooth present, bone, FMPS/FMBS <25%	Systemic disease, pregnant, heavy smokers (>10 cigarettes/day), contralateral tooth missing/heavily restored, periapical lesion >5 mm, mesial/distal bone defects, major bone reconstruction needed	Straumann regular neck, tissue level implants	Zirconia	Cemented (ZrC), Screw‐retained (FCA)	16	16	ZrC: 1 abutment fracture (3%), 1 screw loosening (6%), 1 decementation (3%); FCA: 1 ceramic chipping (3%), 1 screw loosening (6%)	2 mucositis (1 per group)	PES: ZrC 7.0, FCA 7.5; WES: ZrC 7.4, FCA 7.9
Heierle et al. 2019 [21]	Switzerland	Randomized controlled clinical trial	3 years	34	Not reported	Not reported	Single‐tooth implant in anterior maxilla or mandible	Not specified in detail	Straumann bone level implant (SLActive)	Customized zirconia abutments	Cemented: 17, screw‐retained: 17	17	17	CEM: 1 major chipping; SCREW: 1 minor chipping, 1 abutment fracture	Not reported	Not reported
Kraus et al. 2019 [22]	Switzerland	3‐year randomized controlled clinical trial	3 years	44	51.4 ± 17.1 years	22F/22M	Single‐tooth replacement in esthetic zone; informed consent provided	Not specified in detail	OsseoSpeed, DENTSPLY implants	Customized CAD/CAM zirconia abutments	Cemented: 20, screw‐retained: 24	20	24	6 reconstructions lost due to abutment fracture (4 SR, 2 CR), 1 crown removal due to peri‐implantitis (CR)	Peri‐implant mucositis: 2 cases (1 SR, 1 CR); 1 peri‐implantitis in CR group	PD, BoP, PCR, KM measured but no significant group differences
Kraus et al. 2022 [23]	Switzerland	5‐year randomized controlled clinical study	5 years	44	Mean at FU‐5Y: 52.8 ± 17.1 years	22F/22M	Single‐tooth gaps in esthetic zone, no systemic disease, good oral hygiene, informed consent	Not specified in detail	OsseoSpeed, Astra Tech Implant System (Dentsply Sirona)	Customized CAD/CAM zirconia abutments (Atlantis)	Cemented: 20, screw‐retained: 24	20	24	Abutment fractures: 6 (4 SR, 2 CR); minor chipping: 4 (1 SR, 3 CR); screw loosening: 2 (CR only)	Peri‐implant mucositis: 5 (CR only); Peri‐implantitis: 2 (CR only)	BoP: SR 27%, CR 43%; PCR: SR 15%, CR 19%; PD: SR 3.5 mm, CR 3.5 mm
Lamperti et al. 2022 [24]	Switzerland	5‐year randomized controlled clinical trial	5 years	34	55.9 ± 16.0 years (range 28.4–85.4)	Not specified	Single‐tooth implant in anterior maxilla or mandible; 18–80 years old; at least one adjacent tooth; implant position allowing both retention types	Smoking >10 cigarettes/day; poor oral hygiene (PCR >30%); pregnancy	Straumann bone level implant 4.1 mm/3.0 mm SLActive	Customized zirconia abutments	Cemented: 17, screw‐retained: 17	17	17	CEM: 1 major chipping, 4 minor chippings; SCREW: 2 abutment fractures, 1 minor chipping	No peri‐implantitis reported; mucositis not specified	BoP: CEM 37.2%, SCREW 33.3%; PCR: CEM 12.8%, SCREW 6.9%; PD: both groups ~3.2 mm; KT: CEM 3.0 mm, SCREW 3.5 mm
Lv et al. 2023 [25]	China	1‐year randomized controlled clinical trial	1 year	60	AG: 29.9 ± 9.9 years, CG: 32.1 ± 9.8 years	AG: 15M/15F, CG: 15M/15F	≥18 years; single‐implant crown in esthetic region; adjacent to natural teeth; healthy oral condition	Heavy smoking (>10 cigarettes/day), angle >25° between implant and restoration axis, uncontrolled periodontitis, systemic diseases affecting implant therapy, pregnancy	NobelReplace conical connection; NobelActive internal (Nobel biocare)	Angulated screw‐retained: titanium base with zirconia coping; Cemented: prefabricated titanium abutments	Angulated screw‐retained (AG): 30, Cemented (CG): 30	30	30	AG: 1 sealing material loss (3.45%); CG: none	BoP%: AG 21.84% ± 19.97%, CG 37.04% ± 26.28% (*p* = 0.018); no peri‐implantitis	PES/WES: AG 21.71 ± 1.88, CG 21.64 ± 1.87 (*p* = 0.899); PD: AG 2.30 ± 0.68 mm, CG 2.50 ± 0.60 mm
Thoma et al. 2018 [26]	Switzerland	Randomized controlled clinical trial	6 months	33	CEM: median 53.3 years (Q1 = 40.6; Q3 = 62.2), SCREW: median 50.4 years (Q1 = 41.1; Q3 = 57.8)	CEM: 7M/10F, SCREW: 6M/10F	Single‐tooth implants in anterior maxilla or mandible; 18–80 years; adjacent natural teeth; implant position suitable for both retention types	Smoking >15 cigarettes/day, poor oral hygiene (PCR >30%), pregnancy	Straumann bone level implant 3.3 mm/4.1 mm diameter (Straumann, Switzerland)	Customized zirconia abutments	CEM: 17, SCREW: 16	17	16	CEM: 2 minor chippings after insertion; SCREW: none	CEM: more inflammatory cells and periodonto‐pathogens (3 of 4 positive patients); BoP decreased over time	BoP at 6 months: CEM 17%, SCREW 17%; PCR: CEM 11%, SCREW 7%; PD: CEM 3.0 mm, SCREW 3.1 mm
Weigl et al. 2019 [27]	Germany and Estonia	Randomized controlled split‐mouth clinical trial	1 year	22	43 years (range 32–60)	8M/14F	18–70 years; bilateral single‐tooth gaps in premolar/molar region; natural neighboring and antagonist teeth; minor augmentation allowed	Medical contraindications, smoking >5 cigarettes/day, extensive augmentation needed, missing adjacent or antagonist teeth, severe bruxism, poor oral hygiene, pregnancy	Ankylos (Dentsply Sirona Implants, York, USA)	Screw‐retained: monolithic zirconia on titanium base; cemented: porcelain‐fused‐to‐metal crowns on titanium abutments	Cemented: 22, screw‐retained: 22	22	22	CEM: 2 chippings, 2 decementations; SCREW: 1 screw loosening, 2 filling defects	No peri‐implantitis; BOP at 12 months: SCREW 4.5%, CEM 9.1%; PI higher in CEM group but not significant	BoP and PI comparable; no significant differences
Kraus et al. 2024 [28]	Switzerland	Randomized controlled clinical trial	7.5 years	44 at baseline (31 at final follow‐up)	Not explicitly stated (adult patients, mean not reported)	22 female, 22 male	Single‐tooth gap in esthetic zone (maxillary/mandibular anterior, first/second premolars), good general health; some smokers (<10 cigarettes/day), some with treated periodontitis	Heavy smokers (>10 cigarettes/day), untreated periodontitis, systemic conditions not specified but likely excluded	OsseoSpeed, Astra Tech Implant System (Dentsply Sirona, Sweden)	Customized CAD/CAM one‐piece zirconia abutments (Atlantis)	Cement‐retained with veneered lithium disilicate crowns (Ivoclar vivadent) vs. screw‐retained veneered zirconia	19	21	Abutment fracture (7), screw loosening (2), minor chipping (4). Total: 13 (32.5%).	Peri‐implant mucositis (8), peri‐implantitis (1), implant loss (2). Total: 11 (27.5%)	PD ~3 mm (NS), BoP higher in CR (40% vs. 20%, *p* = 0.011), PCR no sig. diff., KM stable

### 3.3. Risk of Bias Assessment

**Figure 2:**
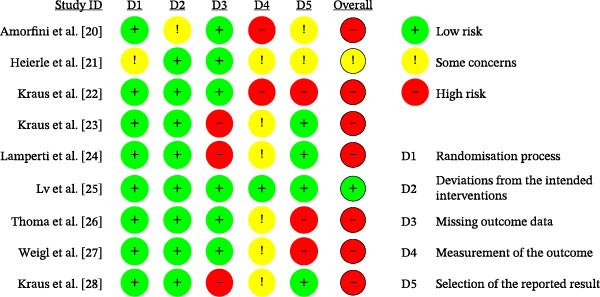
Risk of bias of included randomized controlled trials.

The risk of bias assessment using the Cochrane RoB 2.0 tool revealed that most of the RCTs in this review had some concerns, primarily measurement of outcome and selection of reported outcome. For example, Amorfini et al. [20], Kraus et al. [22, 23], Lamperti et al. [24], Thoma et al. [26], Weigl et al. [27], and Kraus et al. [28] showed a high risk of bias, particularly due to unblinded assessments and selective reporting, which could have influenced subjective results. While only Heierle et al. [21] and Lv et al. [25] had some concerns and low risk. Overall, the evidence suggests that despite some limitations, particularly in the areas of blinding and missing data, the included studies maintained robust randomization processes and appropriate analytical approaches, supporting the reliability of their findings and justifying cautious interpretation of clinical and subjective endpoints (Figure 2).

### 3.4. Effects of Cemented Versus Screw‐Retained Zirconia Single Crowns on Primary Outcomes: MBL, BOP, and PI

**Figure 3:**
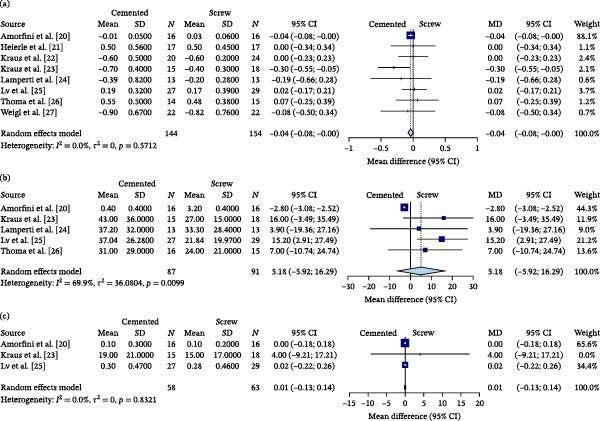
Effects of cemented versus screw‐retained zirconia single crowns on primary outcomes. (a) MBL, (b) BOP, and (c) PI.

In eight RCTs (*n* = 298) [20–27], cemented‐retained zirconia single crowns likely reduce MBL vs. screw‐retained zirconia single crowns (MD −0.04 mm, 95% CI: −0.08 to −0.00; *I* ^2^ = 0%, CoE: moderate) (Figure 3a and Table 2).

**Table 2:** Summary of findings table showing certainty of evidence of effects on outcomes.

Outcomes	Anticipated absolute effects^f^ (95% CI)		Relative effect (95% CI)	No. of participants (studies)	Certainty of the evidence (GRADE)
	Risk with screw‐retained zirconia single crowns	Risk with cemented‐retained zirconia single crowns			
MBL (marginal bone level) Assessed with: mm Follow‐up: range 0.5–10 years	The mean MBL was −0.10 mm	MD 0.04 mm lower (0.08 lower to 0.001 lower)	—	298 (8 RCTs)	⨁⨁⨁◯ Moderate^a^
BoP (bleeding on probing) Assessed with: % Follow‐up: range 0.5–10 years	The mean BoP was 21.8 units	MD 5.18 units higher (5.92 lower to 16.29 higher)	—	178 (5 RCTs)	⨁◯◯◯ Very low^a,b,c^
PI (plaque index) Assessed with: units Follow‐up: range 0.5–10 years	The mean PI was 5.13 units	MD 0.01 units higher (0.13 lower to 0.14 higher)	—	121 (3 RCTs)	⨁⨁◯◯ Low^a,d^
PD (probing depth) Assessed with: mm Follow‐up: range 0.5–10 years	The mean PD was 3.0 units	MD 0.03 units higher (0.16 lower to 0.22 higher)	—	178 (5 RCTs)	⨁⨁⨁◯ Moderate^a^
MPLI (modified plaque index) Assessed with: units Follow‐up: range 0.5–10 years	The mean MPLI was 6.9 units	MD 5.01 units higher (7.05 lower to 17.06 higher)	—	(2 RCTs)	⨁⨁⨁◯ Moderate^a^
KT (keratinized mucosa) Assessed with: mm Follow‐up: range 0.5–10 years	The mean KT was 3.57 mm	MD 0.44 mm lower (1.13 lower to 0.26 higher)	—	56 (2 RCTs)	⨁⨁⨁◯ Moderate^a^
Fracture Follow‐up: range 0.5–10 years	487 per 1000	482 per 1000 (399–580)	RR 0.99 (0.8–1.19)	145 (4 RCTs)	⨁⨁◯◯ Low^a,e^
Loosening Follow‐up: range 0.5–10 years	338 per 1000	318 per 1000 (169–599)	RR 0.94 (0.50–1.77)	119 (3 RCTs)	⨁⨁◯◯ Low^a,e^

*Note:* GRADE working group grades of evidence. High certainty: we are very confident that the true effect lies close to that of the estimate of the effect. Moderate certainty: we are moderately confident in the effect estimate; the true effect is likely to be close to the estimate of the effect, but there is a possibility that it is substantially different. Low certainty: our confidence in the effect estimate is limited; the true effect may be substantially different from the estimate of the effect. Very low certainty: we have very little confidence in the effect estimate; the true effect is likely to be substantially different from the estimate of effect.

Abbreviations: CI, confidence interval; MD, mean difference; RR, risk ratio.

^a^Risk of bias: Each of the included studies showed methodological shortcomings which could have introduced bias (e.g., there was no blinding, incomplete data, or unclear allocation concealment), which weakens confidence in the validity of the findings.

^b^Inconsistency: There was substantial heterogeneity present (*I* ^2^ = 69.9%), suggesting variance across studies in population, intervention, or outcome assessment as contributing to uncertainty of consistent effect.

^c^Imprecision [23, 24, 26]: The confidence intervals were wide and crossed the line of no effect suggesting uncertainty about the size of the true effect (and potential for effect in either direction).

^d^Imprecision [23]: This study, in particular, had wide intervals contributing to additional uncertainty in the pooled estimate.

^e^Imprecision [22, 28]: Again, these studies both showed wide confidence intervals that did not provide a precise estimate of treatment effect, supporting the decision for downgrade because of imprecision.

^f^The risk in the intervention group (and its 95% confidence interval) is based on the assumed risk in the comparison group and the relative effect of the intervention (and its 95% CI).

In five RCTs (*n* = 178) [20, 23–26], cemented‐retained zirconia single crowns had no effect against BoP vs. screw‐retained zirconia single crowns (MD 5.18%, 95% CI: −5.92 to 16.29; *I* ^2^ = 69.9%, CoE: very low) (Figure 3b and Table 2).

In three RCTs (*n* = 121) [20, 23, 25], cemented‐retained zirconia single crowns had no effect against PI vs. screw‐retained zirconia single crowns (MD 0.01 units, 95% CI: −0.13 to 0.14; *I* ^2^ = 0%, CoE: low) (Figure 3c and Table 2).

### 3.5. Effects of Cemented Versus Screw‐Retained Zirconia Single Crowns on Secondary Outcomes: PD, MPLI, KT, Fracture, and Loosening

**Figure 4:**
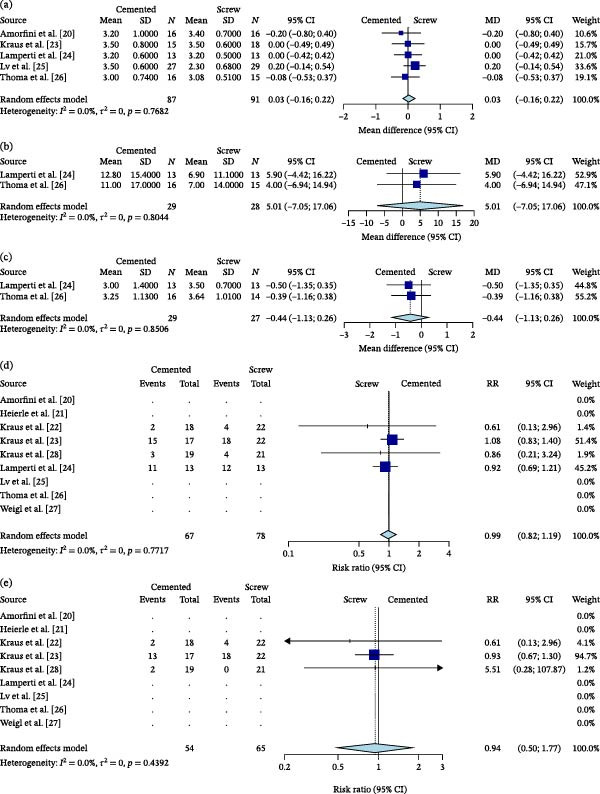
Effects of cemented versus screw‐retained zirconia single crowns on secondary outcomes. (a) PD, (b) MPLI, (c) KT, (d) fracture, and (e) loosening.

In five RCTs (*n* = 178) [20, 23, 24, 26], cemented‐retained zirconia single crowns had no effect against PD vs. screw‐retained zirconia single crowns (MD 0.03 mm, 95% CI: −0.16 to 0.22; *I* ^2^ = 0%, CoE: very low) (Figure 4a).

In two RCTs (*n* = 57) [24, 26], cemented‐retained zirconia single crowns had no effect against MPLI vs. screw‐retained zirconia single crowns (MD 5.01 units, 95% CI: −7.05 to 117.06; *I* ^2^ = 0%, CoE: moderate) (Figure 4b and Table 2).

In two RCTs (*n* = 56) [24, 26], cemented‐retained zirconia single crowns had no effect against KT vs. screw‐retained zirconia single crowns (MD −0.44 mm, 95% CI: −1.13 to 0.26; *I* ^2^ = 0%, CoE: moderate) (Figure 4c and Table 2).

In four RCTs [22–24, 28] (*n* = 135), the analysis of fracture as an outcome in zirconia single crowns showed no significant differences between cemented and screw‐retained restorations (RR = 0.99; 95% CI: 0.82–1.19, CoE: low). These findings suggest that the type of retention does not have a relevant influence on the risk of fracture in this type of crown (Figure 4d and Table 2).

In three RCTs [22, 23, 28] (*n* = 119), the analysis of loosening as an outcome in zirconia single crowns showed no significant differences between cemented and screw‐retained restorations (RR = 0.94; 95% CI: 0.50–1.77, CoE: low). These findings indicate that the type of retention does not significantly affect the risk of loosening in this type of crown (Figure 4e and Table 2).

### 3.6. Subgroup Analyses

The subgroup analyses only showed statistical significance for MBL when evaluated by follow‐up (MD = −0.04 mm; 95% CI: −0.08 to −0.00) and by population (MD = −0.04 mm; 95% CI: −0.08 to −0.00). These findings suggest that, in these specific subgroup conditions, cemented restorations presented slightly less marginal bone loss compared with screw‐retained crowns. On the other hand, the remaining subgroup outcomes did not show significant differences, as the interaction values were nonsignificant (*p* > 0.05) in all cases (Supporting Information S2.1–S2.15).

## 4. Discussion

This systematic review included nine RCTs with 347 patients that tested cemented versus screw‐retained zirconia single crowns. The analysis showed that cemented crowns appeared to reduce the MBL. Otherwise, no statistically significant differences were found for BoP, PI, PD, MPLI, KT, number of fractured crowns, or occurrence of screw loosening between both groups. The CoE for results was ranked from very low to moderate due to concerns regarding imprecision and the risk of bias in all studies.

Our meta‐analysis demonstrated that cemented‐retained crowns presented with significantly lower MBL compared to screw‐retained crowns. This finding appears to contradict the results of a previous systematic review, which found no statistically significant differences between the two retention modalities [29]. The discrepancy is likely explained by differences in inclusion criteria; whereas our analysis was restricted to zirconia crowns, the review included studies with crowns of different materials, such as zirconia and titanium. The inclusion of heterogeneous materials, with potentially distinct biomechanical properties, may have introduced variability that masked the specific effect of the retention method on the MBL.

This finding, which highlights a marginal biological advantage of cemented crowns, gains greater clinical relevance when considering the absence of an associated esthetic disadvantage. The available literature, including the findings of Dini et al. [29], converges in demonstrating no statistically significant differences between cemented and screw‐retained restorations in key esthetic parameters such as the papilla index (MD: −0.01; 95% CI: −0.14 to 0.13; *I* ^2^ = 0%) and the pink esthetic score (MD: −0.02; 95% CI: −0.43 to 0.39; *I* ^2^ = 0%). Therefore, the clinician can opt for cemented retention with the potential benefit of reduced bone loss without concern for compromising the final esthetic outcome.

Because of serious worries about bias and imprecision, the evidence’s level of certainty in this analysis varied from very low to moderate. Future RCTs should make sure that all prespecified outcomes are fully reported and that blinded outcome assessment is used, even though the included studies generally used randomization procedures. However, the majority of them were limited by selective outcome reporting and non‐blinded outcome assessment. The observed imprecision largely resulted from small sample sizes, varying from 22 to 60 participants, highlighting the need for future studies to report detailed sample size calculations that account for potential follow‐up losses and consider the requirements for multiple primary outcomes when applicable [30]. Furthermore, given that the available evidence comes predominantly from European populations and considering reported ethnic variations in dental implant frequency and complication severity [31–33], we recommend expanding research to other geographical regions to enhance the external validation of findings across diverse populations.

The observation that cemented crowns reduce marginal bone loss holds direct clinical relevance for implant prognosis, as established evidence links greater early bone loss to lower long‐term implant survival [34]. This ability to minimize bone loss makes cemented restorations a strategic therapeutic choice, especially in cases where bone preservation is a priority, provided that the primary risk of undetected cement excess is controlled. This risk can be managed by placing the crown margin in an accessible position, an approach supported by scientific evidence [35]. Furthermore, there is evidence that cemented crowns are more cost‐effective compared to screw‐retained crowns [36]. Although our objective was more specific, focusing on zirconia implants, this economic evidence could be extrapolated to our clinical question. However, it is important to consider that the only statistically significant outcome in our analysis was marginal bone loss, and when this small difference is compared to the clinical benchmark of less than 1.5 mm established in the previous literature [37], its clinical importance may be minimal.

Despite a comprehensive, unrestricted search across major databases and manual reference checking, this review may be limited by the potential omission of RCTs from nonindexed sources or the gray literature. Furthermore, the exclusive focus on RCTs, while ensuring high internal validity, limits the assessment of long‐term patient‐reported outcomes, typically captured in real‐world observational studies. Finally, the generalizability and synthesis of findings are constrained by clinical heterogeneity in prosthetic protocols across the included studies.

The greatest limitation of the trials reviewed is that they did not include a clear description or standardization of the method for cementation. The type of cement used was not clearly stated in most studies (e.g., whether it was a temporary cement, such as a dental adhesive or a permanent resin), nor was it clear whether there was a uniform method of cementation used in the same manner across all subjects. There is concern that the method of cementation used will affect the biological and mechanical properties [38, 39] of the cemented zirconia implant. The chemical and physical properties of zirconia (e.g., surface texture, ability to bond with adhesives, and ease of removal of any excess cement) could also have contributed to what were originally observed as mechanical and/or esthetic properties of zirconia implants. Therefore, until then, the interpretation of results from these studies must be conservative; however, there is opportunity for increased recommendations for using zirconia implants, should these options be validated through further research. By addressing these methodological limitations, we would improve the strength of the evidence on the use of zirconia implants, and thus provide improved clinical recommendations for dentists on the use of zirconia implants (Supporting Information S3).

## 5. Conclusion

This systematic review and meta‐analysis indicate that there seems to be slightly less marginal bone loss following the placement of cemented zirconia single crowns compared with screw‐retained zirconia crowns; however, the clinical significance is small. For other biological outcomes including BoP, PI, PD, mucosal parameters, and KT tissue, there were no differences between retention methods. Likewise, prosthetic outcomes such as crown fracture and abutment screw loosening were similar. The overall certainty of the evidence varies from very low‐to‐moderate certainty, primarily due to risk of bias, inconsistency, and imprecision across the studies. Overall, these findings suggest that both cemented and screw‐retained zirconia single crowns can be expected to perform similarly, and the decision of how to retain the single crown can be based on patient factors, clinical feasibility, and prosthetic preference rather than a definable difference in clinical effectiveness.

## Author Contributions

Frank Mayta‐Tovalino, Ivan Calderon‐Cortez, and Daniel Alvitez‐Temoche conceived and designed the study, coordinated the research team, and supervised the overall process, ensuring methodological rigor and scientific integrity. Fran Espinoza‐Carhuancho was responsible for conducting the literature search, extracting the relevant data, and drafting the initial sections of the introduction and discussion. Fran Espinoza‐Carhuancho and Daniel Alvitez‐Temoche contributed to the methodological framework and performed the statistical analyses, preparing the figures and tables that supported the meta‐analysis. Ivan Calderon‐Cortez and Daniel Alvitez‐Temoche participated in the validation of the included studies, curated the data, and assisted in the editing of the manuscript. Miguel Cabanillas‐Lazo prepared the original draft, formatted the references, and ensured that the manuscript complied with the journal’s editorial requirements. Joshuan J. Barboza and Frank Mayta‐Tovalino carried out the formal analysis, interpreted the results, and adapted the manuscript bilingually to enhance its accessibility. Adrian V. Hernandez provided expertise in meta‐analysis methodology, supervised the advanced statistical procedures, and gave final approval to the manuscript.

## Funding

No funding was received for this manuscript.

## Ethics Statement

The authors have nothing to report.

## Consent

The authors have nothing to report.

## Conflicts of Interest

The authors declare no conflicts of interest.

## Supporting Information

Additional supporting information can be found online in the Supporting Information section.

## Supporting information


**Supporting Information** Additional data and figures supporting the systematic review and meta‐analysis on the clinical efficacy of cemented versus screw‐retained zirconia single crowns.

## Data Availability

The data are available upon request to the corresponding author.

## References

[1] Jung R. E. , Zembic A. , Pjetursson B. E. , Zwahlen M. , and Thoma D. S. , Systematic Review of the Survival Rate and the Incidence of Biological, Technical, and Aesthetic Complications of Single Crowns on Implants Reported in Longitudinal Studies with a Mean Follow-up of 5 Years, Clinical Oral Implants Research. (2012) 23, no. Suppl, 6, 2–21. 23062124 10.1111/j.1600-0501.2012.02547.x

[2] Pjetursson B. E. , Thoma D. , Jung R. , Zwahlen M. , and Zembic A. , A Systematic Review of the Survival and Complication Rates of Implant-Supported Fixed Dental Prostheses (FDPs) after a Mean Observation Period of at least 5 Years, Clinical Oral Implants Research. (2012) 23, no. Suppl, 6, 22–38. 10.1111/j.1600-0501.2012.02546.x 23062125

[3] Pjetursson B. E. , Valente N. A. , Strasding M. , Zwahlen M. , Liu S. , and Sailer I. , A Systematic Review of the Survival and Complication Rates of Zirconia-Ceramic and Metal-Ceramic Single Crowns, Clinical Oral Implants Research. (2018) 29, no. Suppl, 16, 199–214. 30328190 10.1111/clr.13306

[4] Shokry M. , Al-Zordk W. , and Ghazy M. , Retention Strength of Monolithic Zirconia Crowns Cemented With Different Primer-Cement Systems, BMC Oral Health. (2022) 22, no. 1, 10.1186/s12903-022-02223-0. PMC9118569 35590310

[5] Rabel K. , Spies B. C. , Pieralli S. , Vach K. , and Kohal R. J. , The Clinical Performance of All-Ceramic Implant-Supported Single Crowns: A Systematic Review and Meta-Analysis, Clinical Oral Implants Research. (2018) 29, no. Suppl, 18, 196–223. 30306684 10.1111/clr.13337

[6] Sailer I. , Philipp A. , Zembic A. , Pjetursson B. E. , Hämmerle C. H. , and Zwahlen M. , A Systematic Review of the Performance of Ceramic and Metal Implant Abutments Supporting Fixed Implant Reconstructions, Clinical Oral Implants Research. (2009) 20, no. Suppl, 4, 4–31. 19663946 10.1111/j.1600-0501.2009.01787.x

[7] Scarano A. , Lorusso F. , Orsini T. , Morra M. , Iviglia G. , and Valbonetti L. , Biomimetic Surfaces Coated with Covalently Immobilized Collagen Type I: An X-Ray Photoelectron Spectroscopy, Atomic Force Microscopy, Micro-CT and Histomorphometrical Study in Rabbits, International Journal of Molecular Sciences. (2019) 20, no. 3, 10.3390/ijms20030724. PMC6387268 30744023

[8] Scarano A. , Carinci F. , and Lorusso F. , et al.Ultrasonic vs Drill Implant Site Preparation: Post-Operative Pain Measurement Through VAS, Swelling and Crestal Bone Remodeling: A Randomized Clinical Study, Materials. (2018) 11, no. 12, 10.3390/ma11122516, 2516. 30544962 PMC6316965

[9] Mangano C. , Piattelli A. , and Scarano A. , et al.A Light and Scanning Electron Microscopy Study of Human Direct Laser Metal Forming Dental Implants, The International Journal of Periodontics & Restorative Dentistry. (2014) 34, no. 1, e9–e17, 10.11607/prd.1213. 24396851

[10] Gomes É.A. , Tiossi R. , Faria A. C. , Rodrigues R. C. , and Ribeiro R. F. , Torque Loss Under Mechanical Cycling of Long-Span Zirconia and Titanium-Cemented and Screw-Retained Implant-Supported CAD/CAM Frameworks, Clinical Oral Implants Research. (2014) 25, no. 12, 1395–1402, 10.1111/clr.12286. 25539006

[11] Nissan J. , Narobai D. , Gross O. , Ghelfan O. , and Chaushu G. , Long-Term Outcome of Cemented Versus Screw-Retained Implant-Supported Partial Restorations, The International Journal of Oral & Maxillofacial Implants. (2011) 26, no. 5, 1102–1107. 22010095

[12] Lewis S. , Beumer J.III, Hornburg W. , and Moy P. , The “UCLA” Abutment, International Journal of Oral & Maxillofacial Implants. (1988) 3, no. 3, 183–189. 3074050

[13] Michalakis K. X. , Hirayama H. , and Garefis P. D. , Cement-Retained Versus Screw-Retained Implant Restorations: A Critical Review, The International journal of oral & maxillofacial implants. (2003) 18, no. 5, 719–728. 14579961

[14] Karl M. , Taylor T. D. , Wichmann M. G. , and Heckmann S. M. , In Vivo Stress Behavior in Cemented and Screw-Retained Five-Unit Implant FPDs, Journal of Prosthodontics-Implant Esthetic and Reconstructive Dentistry. (2006) 15, no. 1, 20–24, 10.1111/j.1532-849X.2006.00064.x. 16433647

[15] Korsch M. , Obst U. , and Walther W. , Cement-Associated Peri-Implantitis: A Retrospective Clinical Observational Study of Fixed Implant-Supported Restorations Using a Methacrylate Cement, Clinical Oral Implants Research. (2014) 25, no. 7, 797–802, 10.1111/clr.12173. 23600620

[16] Scarano A. , Stoppaccioli M. , and Casolino T. , Zirconia Crowns Cemented on Titanium Bars Using CAD/CAM:A 5-Year Follow-up Prospective Clinical Study of 9 patients, BMC Oral Health. (2019) 19, no. 1, 10.1186/s12903-019-0988-x. PMC6921470 31856799

[17] Page M. J. , McKenzie J. E. , and Bossuyt P. M. , et al.The PRISMA 2020 Statement: An Updated Guideline for Reporting Systematic Reviews, British Medical Journal. (2021) 372. 10.1136/bmj.n71 PMC8005924 33782057

[18] Thorlund K. , Imberger G. , and Johnston B. C. , et al.Evolution of Heterogeneity (I2) Estimates and Their 95% Confidence Intervals in Large Meta-Analyses, PLoS One. (2012) 7, no. 7, 10.1371/journal.pone.0039471, e39471. 22848355 PMC3405079

[19] Brozek J. L. , Canelo-Aybar C. , and Akl E. A. , et al.GRADE Guidelines 30: The GRADE Approach to Assessing the Certainty of Modeled Evidence-An Overview in the Context of Health Decision-Making, Journal of Clinical Epidemiology. (2021) 129, 138–150, 10.1016/j.jclinepi.2020.09.018. 32980429 PMC8514123

[20] Amorfini L. , Storelli S. , Mosca D. , Scanferla M. , and Romeo E. , Comparison of Cemented vs Screw-Retained, Customized Computer-Aided Design/Computer-Assisted Manufacture Zirconia Abutments for Esthetically Located Single-Tooth Implants: A 10-Year Randomized Prospective Study, The International Journal of Prosthodontics. (2018) 31, no. 4, 359–366, 10.11607/ijp.5305. 29624628

[21] Heierle L. , Wolleb K. , Hämmerle C. H. , Wiedemeier D. B. , Sailer I. , and Thoma D. S. , Randomized Controlled Clinical Trial Comparing Cemented Versus Screw-Retained Single Crowns on Customized Zirconia Abutments: 3-Year Results, The International Journal of Prosthodontics. (2019) 32, no. 2, 174–176, 10.11607/ijp.6080. 30856641

[22] Kraus R. D. , Epprecht A. , Hämmerle C. H. F. , Sailer I. , and Thoma D. S. , Cemented vs Screw-Retained Zirconia-Based Single Implant Reconstructions: A. 3-Year Prospective Randomized Controlled Clinical Trial, Clinical Implant Dentistry and Related Research. (2019) 21, no. 4, 578–585, 10.1111/cid.12735. 30861635

[23] Kraus R. D. , Espuelas C. , Hämmerle C. H. F. , Jung R. E. , Sailer I. , and Thoma D. S. , Five-Year Randomized Controlled Clinical Study Comparing Cemented and Screw-Retained Zirconia-Based Implant-Supported Single Crowns, Clinical Oral Implants Research. (2022) 33, no. 5, 537–547, 10.1111/clr.13913. 35224774 PMC9313572

[24] Lamperti S. T. , Wolleb K. , Hämmerle C. H. F. , Jung R. E. , Hüsler J. , and Thoma D. S. , Cemented Versus Screw-Retained Zirconia-Based Single-Implant Restorations: 5-Year Results of a Randomized Controlled Clinical Trial, Clinical Oral Implants Research. (2022) 33, no. 4, 353–361, 10.1111/clr.13895. 35051314 PMC9305781

[25] Lv X. , Pu Y. , and Zhang X. , et al.One-Piece Versus Two-Piece Zirconia Abutment Supported Single Implant Crown in the Esthetic Region: 3-Year Results From a Split-Mouth Randomized Controlled Clinical Trial, Clinical Oral Implants Research. (2023) 34, no. 12, 1330–1341, 10.1111/clr.14173. 37655630

[26] Thoma D. S. , Sailer I. , Mühlemann S. , Gil A. , Jung R. E. , and Hämmerle C. H. F. , Randomized Controlled Clinical Study of Veneered Zirconia Abutments for Single Implant Crowns: Clinical, Histological, and Microbiological Outcomes, Clinical Implant Dentistry and Related Research. (2018) 20, no. 6, 988–996, 10.1111/cid.12674. 30328283

[27] Weigl P. , Saarepera K. , Hinrikus K. , Wu Y. , Trimpou G. , and Lorenz J. , Screw-Retained Monolithic Zirconia vs. Cemented Porcelain-Fused-to-Metal Implant Crowns: A Prospective Randomized Clinical Trial in Split-Mouth Design, Clinical Oral Investigations. (2019) 23, no. 3, 1067–1075, 10.1007/s00784-018-2531-x. 29946832

[28] Kraus R. D. , Hjerppe J. , Naenni N. , Balmer M. , Jung R. E. , and Thoma D. S. , 7.5-Year Randomized Controlled Clinical Study Comparing Cemented and Screw-Retained One-Piece Zirconia-Based Implant-Supported Single Crowns, Clinical Oral Implants Research. (2024) 35, no. 12, 1669–1675, 10.1111/clr.14346. 39172056 PMC11629449

[29] Dini C. , Borges G. A. , Costa R. C. , Magno M. B. , Maia L. C. , and Barão V. A. R. , Peri-Implant and Esthetic Outcomes of Cemented and Screw-Retained Crowns Using Zirconia Abutments in Single Implant-Supported Restorations-A Systematic Review and Meta-Analysis, Clinical Oral Implants Research. (2021) 32, no. 10, 1143–1158, 10.1111/clr.13824. 34352144

[30] Ying X. , Freedland K. E. , Powell L. H. , Stuart E. A. , Ehrhardt S. , and Mayo-Wilson E. , Determining Sample Size for Pilot Trials: A Tutorial, British Medical Journal. (2025) 390, e083405. 40780848 10.1136/bmj-2024-083405

[31] Weatherspoon D. J. , Chen H. , and Dye B. A. , Implant and Implant Restoration Trends Among Adults 50 Years and Older in the United States, National Health and Nutrition Examination Survey 1999-2020, The Journal of the American Dental Association. (2024) 155, no. 7, 574–586.e3, 10.1016/j.adaj.2024.03.005. 38804988

[32] Okunseri C. , Bajorunaite R. , Matthew R. , and Iacopino A. M. , Racial and Ethnic Variation in the Provision of Dental Procedures, Journal of Public Health Dentistry. (2007) 67, no. 1, 20–27, 10.1111/j.1752-7325.2007.00004.x. 17436975

[33] Nath S. , Sethi S. , and Bastos J. L. , et al.The Global Prevalence and Severity of Dental Caries Among Racially Minoritized Children: A Systematic Review and Meta-Analysis, Caries Research. (2023) 57, no. 4, 485–508, 10.1159/000533565. 37734332

[34] Sommer M. , Zimmermann J. , Grize L. , and Stübinger S. , Marginal Bone Loss 1 Year After Implantation: A Systematic Review of Different Loading Protocols, International Journal of Oral and Maxillofacial Surgery. (2020) 49, no. 1, 121–134, 10.1016/j.ijom.2019.03.965. 31255443

[35] Linkevicius T. , Vindasiute E. , Puisys A. , and Peciuliene V. , The Influence of Margin Location on the Amount of Undetected Cement Excess After Delivery of Cement-Retained Implant Restorations, Clinical Oral Implants Research. (2011) 22, no. 12, 1379–1384, 10.1111/j.1600-0501.2010.02119.x. 21382089

[36] Ramamoorthi M. and Esfandiari S. , Cement-Retained Implant-Supported Prosthesis in Partially Edentulous Patients: An Oral Health Technology Assessment Report, JDR Clinical & Translational Research. (2016) 1, no. 1, 40–50, 10.1177/2380084416634071. 30931699

[37] Albrektsson T. , Zarb G. , Worthington P. , and Eriksson A. R. , The Long-Term Efficacy of Currently Used Dental Implants: A Review and Proposed Criteria of Success, The International Journal of Oral & Maxillofacial Implants. (1986) 1, no. 1, 11–25. 3527955

[38] Salvi G. E. , Monje A. , and Tomasi C. , Long-Term Biological Complications of Dental Implants Placed Either in Pristine or in Augmented Sites: A Systematic Review and Meta-Analysis, Clinical Oral Implants Research. (2018) 29, no. Suppl, 16, 294–310. 30328184 10.1111/clr.13123

[39] Fiorillo L. , D’Amico C. , Ronsivalle V. , Cicciù M. , and Cervino G. , Single Dental Implant Restoration: Cemented or Screw-Retained? A Systematic Review of Multi-Factor Randomized Clinical Trials, Prosthesis. (2024) 6, no. 4, 871–886, 10.3390/prosthesis6040063.

